# Long-tip high-speed atomic force microscopy for nanometer-scale imaging in live cells

**DOI:** 10.1038/srep08724

**Published:** 2015-03-04

**Authors:** Mikihiro Shibata, Takayuki Uchihashi, Toshio Ando, Ryohei Yasuda

**Affiliations:** 1 Max Planck Florida Institute for Neuroscience, Jupiter, FL 33458, USA; 2 Department of Neurobiology, Duke University Medical School, Durham, NC 27710, USA; 3 Department of Physics, Kanazawa University, Kanazawa 920-1192, Japan; 4 Bio-AFM Frontier Research Center, Kanazawa University, Kanazawa 920-1192, Japan; 5 CREST/JST, Tokyo 102-0075, Japan

## Abstract

Visualization of morphological dynamics of live cells with nanometer resolution under physiological conditions is highly desired, but challenging. It has been demonstrated that high-speed atomic force microscopy is a powerful technique for visualizing dynamics of biomolecules under physiological conditions. However, application of high-speed atomic force microscopy for imaging larger objects such as live mammalian cells has been complicated because of the collision between the cantilever and samples. Here, we demonstrate that attaching an extremely long (~3 μm) and thin (~5 nm) tip by amorphous carbon to the cantilever allows us to image the surface structure of live cells with the spatiotemporal resolution of nanometers and seconds. We demonstrate that long-tip high-speed atomic force microscopy is capable of imaging morphogenesis of filopodia, membrane ruffles, pit formation, and endocytosis in COS-7, HeLa cells and hippocampal neurons.

Atomic force microscopy (AFM) can image surface topography of objects with the resolution of single atoms on solid surface^1^. Furthermore, since AFM imaging can be performed in an aqueous solution, it has been applied to biological samples such as proteins, nucleic acids, membrane lipids and even live cells under physiological conditions^2^,^3^,^4^,^5^,^6^,^7^. AFM is also used for force measurements to estimate the strength of intra- and intermolecular bonds at the single molecule level^8^,^9^ and the elasticity of biological objects^10^,^11^. However, the use of conventional AFM for imaging dynamics of live biological samples has been difficult, since it takes many minutes to acquire an image and a tip motion often damages the sample.

To apply AFM to dynamic biological system, the speed of AFM scanning has been extensively optimized by modifying each component of AFM^12^,^13^. The resulting high-speed AFM (HS-AFM) achieved the scanning speed orders of magnitudes faster than that of conventional AFM, opening a way to monitor the conformational dynamics of single proteins on substrates with a temporal resolution of subseconds. To avoid damaging biological samples, HS-AFM uses a tapping mode scanning system combined with an extremely soft cantilever (100–200 pN/nm). In the past few years, this technique realized various dynamic processes of biological samples including photo-induced conformational change of bacteriorhodopsin^14^,^15^,^16^, myosin V walking on an actin filament^17^ and rotary catalysis of F_1_-ATPase^18^, reaction processes of DNA targeting enzymes^19^,^20^, nucleosome dynamics^21^,^22^ and local conformational changes of DNA strands^23^,^24^. However, applications of this technique to imaging nano-structure of live mammalian cells has been complicated since the length scale of mammalian cells is orders of magnitude larger than that of proteins.

In order to further optimize HS-AFM for cellular imaging, we previously developed HS-AFM with a wide-area scanner, and demonstrated that HS-AFM is capable of imaging large area of mammalian cells (up to ~5 × 5 μm^2^) with temporal resolution of seconds^25^. In addition, HS-AFM has been combined with optical microscopy to facilitate the positioning of the scanning tip to samples^26^,^27^,^28^. However, because of the collision between a cantilever and cells, it was difficult to image cell surface with high spatial resolution and long-term imaging often caused severe damages to the cells.

In this paper, we further improved HS-AFM by fabricating an extremely long tip on the cantilever (long-tip HS-AFM or LT-HS-AFM), and demonstrate that this modification enables us to image nanometer-scale morphogenesis in mammalian cells and hippocampal neurons for more than tens of minutes without damages. In particular, we show that LT-HS-AFM makes analyses of cellular morphogenesis in response to extracellular stimulation and pharmacological intervention possible.

## Results

**Figure 1:**
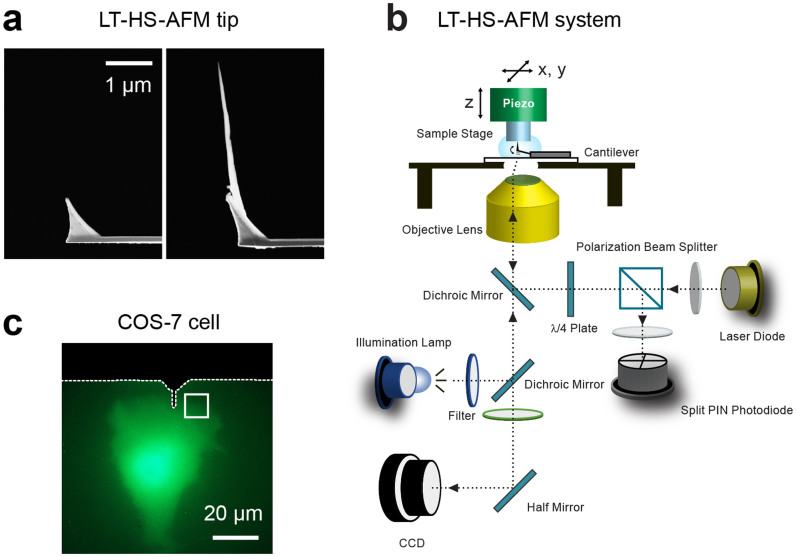
LT-HS-AFM system combined with fluorescence microscopy. (a) Scanning electron microscopy (SEM) images of the end of the cantilever without an electron-beam-deposit (EBD) tip (left) and with ~3 μm EBD tip (right). (b) A schematic illustration of LT-HS-AFM system. An arc lamp for fluorescence excitation and a CCD camera to capture bright-field and fluorescence images were installed under the LT-HS-AFM system. (c) Fluorescence images of a COS-7 cell transfected with mEGFP. The white broken lines show the shadow of the cantilever. The area indicated with the white square was subjected to LT-HS-AFM imaging. LT-HS-AFM images corresponding to c is shown in Figure 2.

We optimized HS-AFM for live-cell imaging by making following modifications. First, we fabricated an extremely long (~3 μm), and thin (5–8 nm in diameter) tip by amorphous carbon grown vertically on top of a soft cantilever (100–200 pN/nm) of AFM by using a phenol deposition technique under a scanning electron microscope^29^ (Fig. 1a). This new tip design enables imaging of cells with high spatial resolution with minimum collision to the cell when scanned under a tapping-mode (~100 nm free amplitude, 800 kHz). Also, we adapted a wide-area, high speed piezo scanner, which has the maximum scan rage of 40 × 40 μm^2^ and is capable of scanning ~5 × 5 μm^2^ area within ~5 s (Ref. 25). In addition, to facilitate locating the long-tip to the target area, we combined LT-HS-AFM with fluorescence microscopy (Fig. 1b,c and Supplementary Fig. 1).

**Figure 2:**
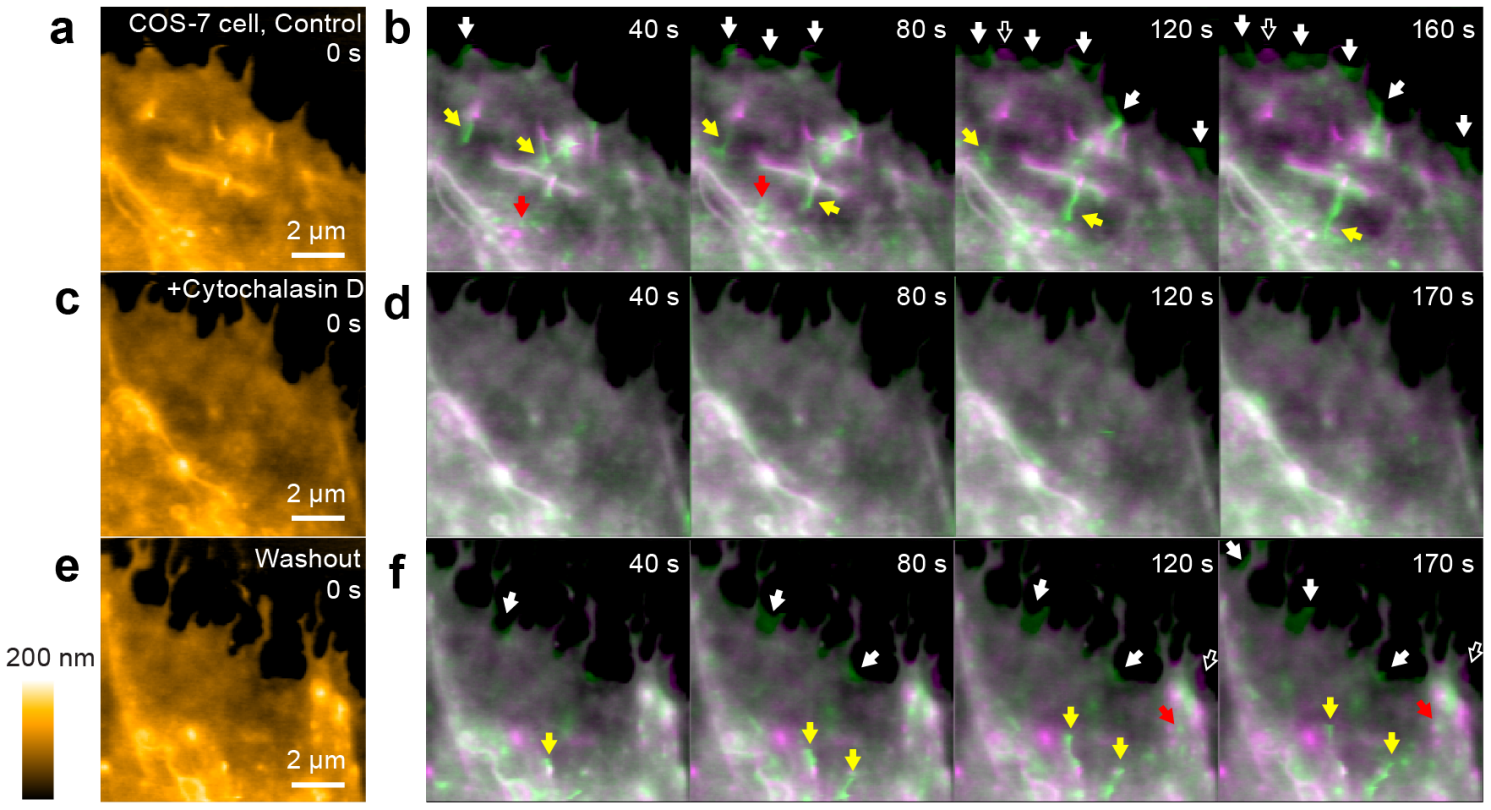
LT-HS-AFM images of a living COS-7 cell. (a) A LT-HS-AFM topographical image acquired from the area indicated in Figure 1c before the addition of cytochalasin D. The corresponding movie (10 s per frame) is in Supplementary Movie 1. (b) LT-HS-AFM images taken at the indicated times (green) overlaid with the image at 0 s (magenta). Filled and open white arrows indicate newly appeared and disappeared structures at the leading edge of the cell, respectively. Yellow and red arrows indicate the movement of protrusions and vesicles, respectively. (c-f) LT-HS-AFM image after the application of cytochalasin D (20 ng/mL) (c, d) and following washout (e, f). See also Supplementary Movies 1 and 2.

Using LT-HS-AFM, we first imaged a COS-7 cell transfected with mEGFP (Fig. 2). We placed the cantilever tip at the leading edge of the cell using fluorescence microscopy (Fig. 1c) and scanned over ~10 × 10 μm^2^ area to observe the dynamics of the surface topology of the cell at 10 s per frame. LT-HS-AFM imaging of the cell could be performed for more than 1 hour without any obvious cellular damage. At the leading edge, the LT-HS-AFM movie showed membrane ruffling and extension and retraction of filopodia over a few seconds (Fig. 2a, b and Supplementary Movie 1). After the application of an inhibitor of actin polymerization, cytochalasin D^30^, these structural dynamics were abolished (Fig. 2c, d and Supplementary Movie 1). Subsequently, after the cell was washed out for ~30 min with the imaging solution, the dynamic movement of the leading edge was recovered (Fig. 2e, f and Supplementary Movie 2). These results indicate that the observed membrane dynamics requires actin polymerization.

**Figure 3:**
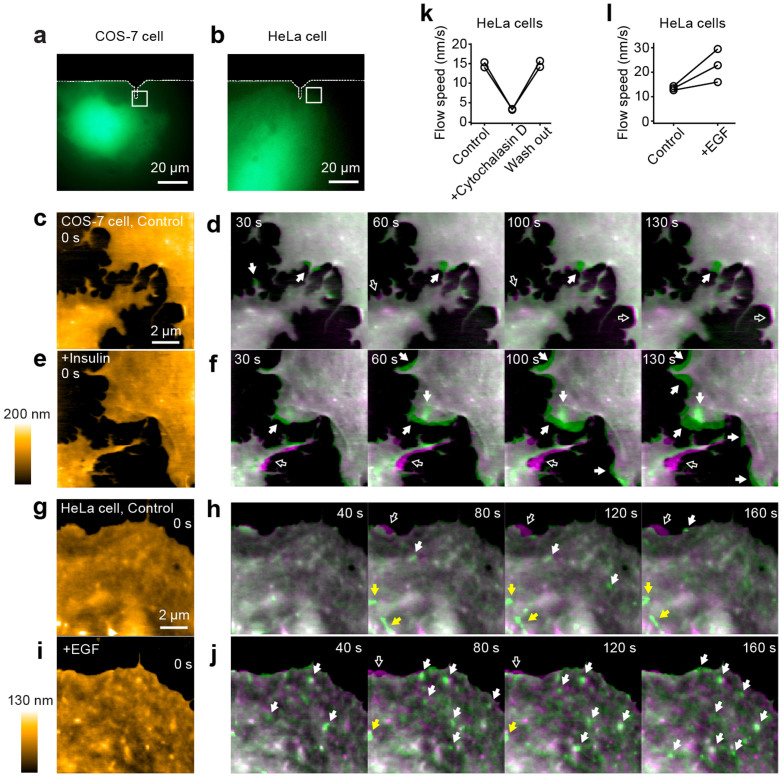
LT-HS-AFM images of living COS-7 and HeLa cells in response to extracellular stimuli. (a, b) Fluorescence image of a COS-7 and HeLa cell transfected with mEGFP. The white dotted lines indicate the shadow of the cantilever. The white square corresponding to the LT-HS-AFM scanning area. LT-HS-AFM images corresponding to a and b are shown in c-f and g-j, respectively. (c, e) A LT-HS-AFM topographical image of the same cell as in a before (c) and after (e) insulin application (20 μg/mL). Cells were starved with serum-free medium for ~1 hour before the experiments. (d, f) LT-HS-AFM images taken at the indicated times (green) overlaid with the image at 0 s (magenta) before (d) and after (f) insulin application. Filled and open white arrows indicate newly appeared and disappeared structures at the leading edge of the cell, respectively. The corresponding movie (10 s per frame) is in Supplementary Movie 3. (g, i) A LT-HS-AFM topographical image of the same cell as in b before (g) and after (i) EGF application (20 ng/mL). (h, j) LT-HS-AFM images taken at the indicated times (green) overlaid with the image at 0 s (magenta) before (h) and after (j) EGF application. The corresponding movie (10 s per frame) is in Supplementary Movie 4. (k) Flow speed of retrograde actin flow at the leading edge of HeLa cells observed by LT-HS-AFM before and after application of cytochalasin D. (mean ± s.d.) (l) Flow speed of retrograde actin flow at the leading edge of HeLa cells observed by LT-HS-AFM before and after EGF application.

It is known that membrane dynamics are regulated by growth factors and hormones^31^. Thus, we imaged the leading edge of COS-7 cells before and after application of insulin (Fig. 3a). We found that, in response to insulin application, the ruffling at the leading edge was enhanced (Fig. 3c–f and Supplementary Movie 3). In addition, we imaged the leading edge of HeLa cells (Fig. 3b), since it is known that HeLa cells display a retrograde actin flow at the leading edge^32^. Consistent with the previous reports^32^, we observed relatively homogeneous flow of the membrane with speeds of 14.7 ± 0.90 nm/s (before), 3.29 ± 0.064 nm/s (after cytochalasin D) and 14.9 ± 1.1 nm/s (washout) with LT-HS-AFM (Fig. 3g, h, k and Supplementary Movie 4). Compared to COS-7 cells, the membrane movement was much more directional and uniform. When EGF was applied to the cell, the averaged membrane flow was accelerated from 13.5 ± 0.83 nm/s (before) to 22.8 ± 6.8 nm/s (after EGF) (Fig. 3i, j, l and Supplementary Movie 4). Thus, these results demonstrate that LT-HS-AFM is capable of imaging ligand-induced membrane morphogenesis.

**Figure 4:**
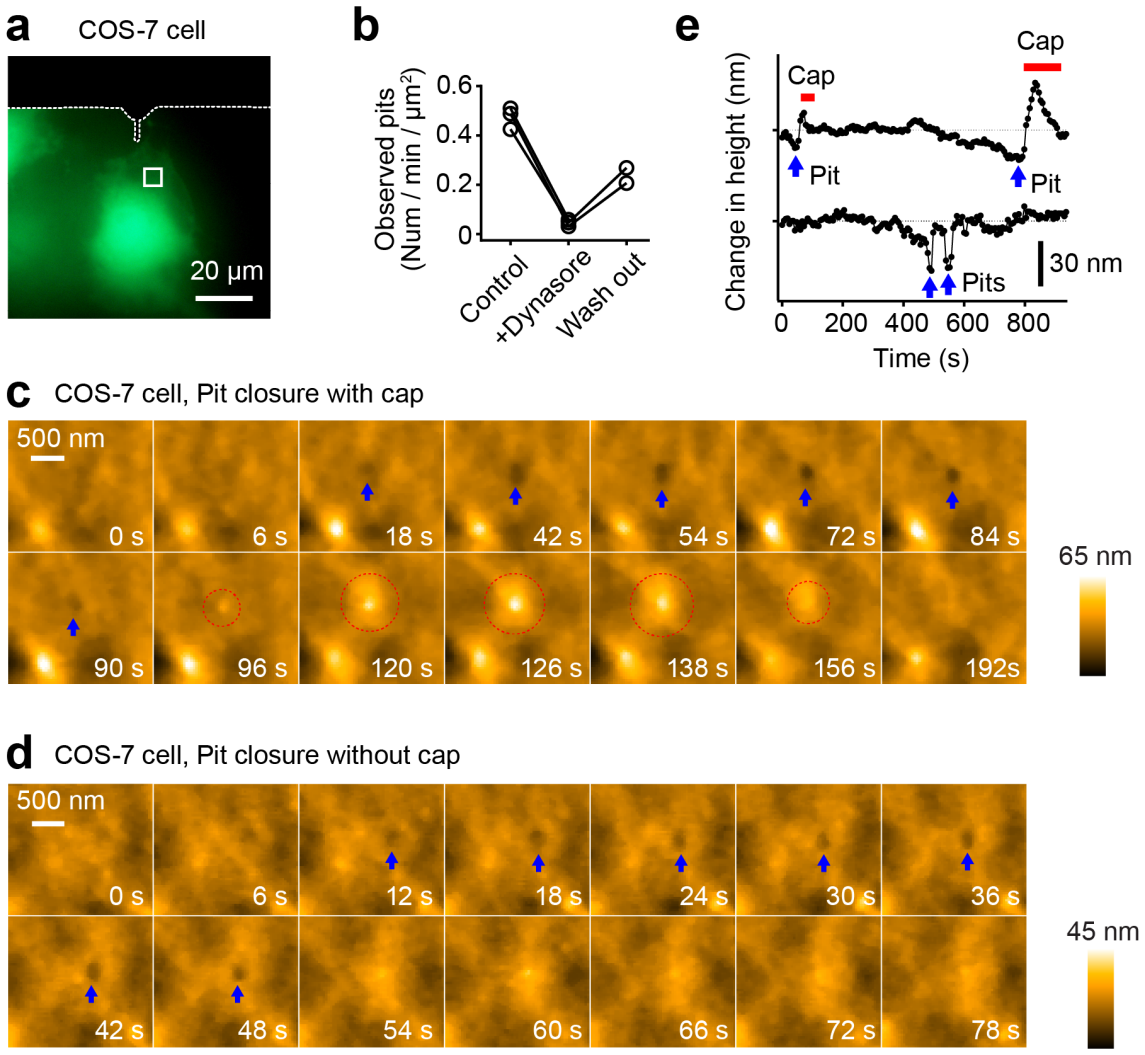
Pits formation on the plasma membrane of a living COS-7 cell. (a) Fluorescence images of a COS-7 cells transfected with mEGFP. The white broken lines show the shadow of the cantilever. The area indicated with the white square was subjected to LT-HS-AFM imaging. (b) The number of observed pits per min per μm^2^ area before, after and washout of dynasore application. (c) A sequence of magnified LT-HS-AFM images of a living COS-7 cell, taken at 6 s per frame, during the pit formation and the closure of the pit with a cap. Blue arrows indicate the formation of the pit. Dotted red circles indicate the formation of the closure cap. (d) A sequence of magnified LT-HS-AFM images during pit formation and closure of a pit without a cap. Blue arrows indicate the formation of the pit. (e) Time courses of the depth of pits with and without closure caps. More data are shown in Supplementary Figure 4. Red bars indicate the formation of closure caps. Blue arrows indicate pit formation.

Next, we observed the membrane dynamics near the nucleus of COS-7 cells (Fig. 4a). In this area, we observed pits cycling between the open and closed forms on the same area of the plasma membrane (0.474 ± 0.044 pits/min/μm^2^; Supplementary Fig. 2 and Supplementary Movie 5). After the application of dynasore, an inhibitor of dynamin^33^, these pit dynamics were abolished (0.0454 ± 0.014 pits/min/μm^2^). Subsequently, the dynamics of pits were partially recovered by washing out the drug for ~30 min (0.238 ± 0.044 pits/min/μm^2^; Fig. 4b, Supplementary Fig. 2 and Supplementary Movies 5, 6). These results suggest that the closure of the pits reflects dynamin-dependent endocytosis. Further, we often observed “cap-type” endocytosis, in which pits are closed by protrusions formed near the pits^27^,^34^ (Fig. 4c–e). When we overexpressed the constitutive active mutant of Rab5, which positively regulates endocytosis^35^,^36^, the lifetime of pits became shorter (31.5 ± 0.5 s for cells expressing mEGFP and 14.4 ± 0.3 s for cells expressing mEGFP-Rab5(Q79L)), further suggesting that the pits formation and closure are the processes of endocytosis (Supplementary Fig. 3 and Supplementary Movie 7).

**Figure 5:**
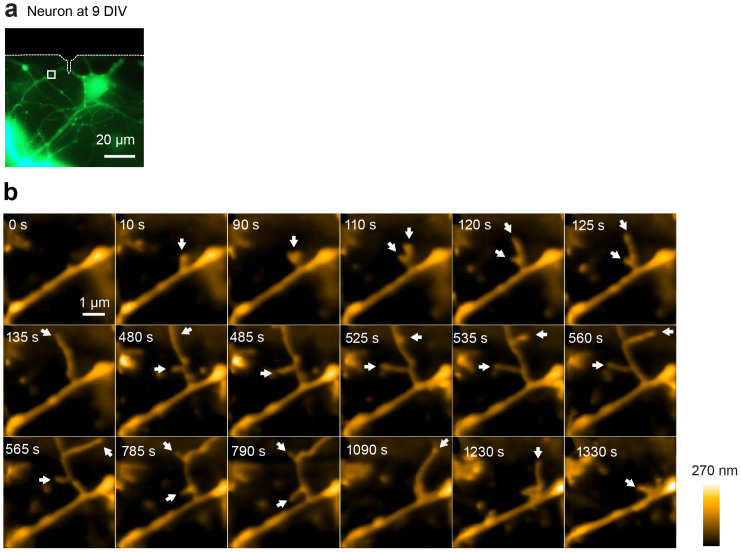
LT-HS-AFM images of a living cultured hippocampal neuron at 9 days *in vitro*. (a) Fluorescence image of a hippocampal neuron transfected with mEGFP. The white dotted lines show the shadow of the cantilever. The white square indicates the LT-HS-AFM imaging area. (b) A sequence of LT-HS-AFM topographical images of a dendrite captured from the area indicated in a. The white arrows indicate the growth of filopodia. The size of the dendrite is ~400 nm with ~200 nm height. The corresponding movie is in Supplementary Movie 8.

**Figure 6:**
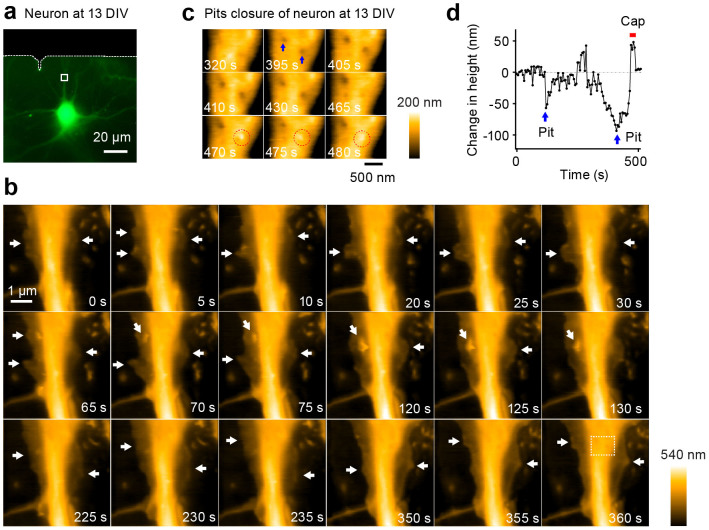
LT-HS-AFM images of a living cultured hippocampal neuron at 13 days *in vitro*. (a) Fluorescence image of a hippocampal neuron transfected with mEGFP. Corresponding LT-HS-AFM images for a is shown in b. (b) A sequence of LT-HS-AFM topographical images at 5 s per frame. White arrows indicate the thin, sheet-like ruffling structure. White dotted box at 360 s indicates the magnified region shown in c. The corresponding LT-HS-AFM movie is shown in Supplementary Movie 9. (c) A sequence of magnified LT-HS-AFM images during the formation and the closure of pits. Blue arrows indicate the pit formation and dotted red circles indicate the formation of the closure cap. (d) Time course of the depth and height of the pit with and without the closure cap. Red bars indicate the formation of the closure cap. Blue arrows indicate pit formations.

**Figure 7:**
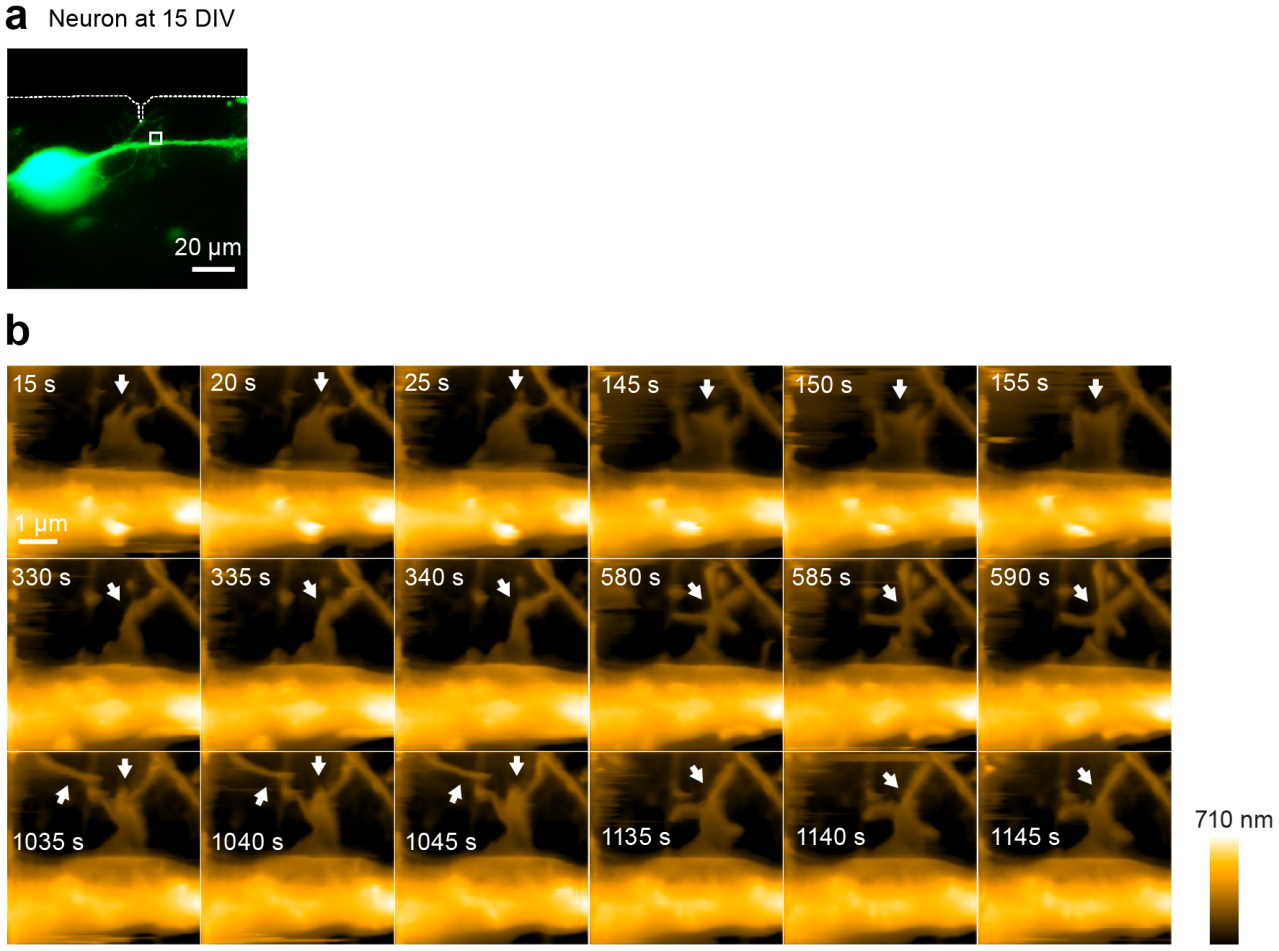
LT-HS-AFM images of a living cultured hippocampal neuron at 15 days *in vitro*. (a) Fluorescence image of a hippocampal neuron transfected with mEGFP. Corresponding LT-HS-AFM images for a is shown in b. (b) A sequence of LT-HS-AFM topographical images at 5 s per frame. White arrows indicate the dynamics of spine-like structure. The corresponding LT-HS-AFM movie is shown in Supplementary Movie 10.

Next we applied our LT-HS-AFM to visualize morphological changes of live neurons. We plated hippocampal neurons from P0 rats at low density on top of a layer of glia cells placed on a glass stage and cultured for 9–15 days^37^,^38^. We identified a neuron transfected with mEGFP using fluorescence imaging and acquired high-resolution topographical images using LT-HS-AFM (Fig. 5a, 6a, 7a). On thin dendrites, we often observed rapid filopodia extension and retraction (Fig. 5b and Supplementary Movie 8). Also, around dendritic shafts, we observed a thin and sheet-like ruffling structure on top of glia cells. This structure would be difficult to image under an optical microscope due to its small width (~150–500 nm wide) and thickness (~70 nm high), which are beyond the resolution of optical microscopes (Fig. 6b and Supplementary Movie 9). Pits undergoing cycles between open and closed states were also observed on the dendritic surface (Fig. 6c, d). These events presumably indicate spontaneous endocytosis events. Some of the closure events were associated with cap formations similarly to endocytosis observed in COS-7 cells. Notably, in relatively mature neurons at 15 days in vitro (DIV), we succeeded in imaging protrusions from dendrites that appear to be dendritic spines. These images revealed highly dynamic morphogenesis of the spine-like structure over the time scale of seconds (Fig. 7b and Supplementary Movie 10).

## Discussion

As demonstrated above, the development of LT-HS-AFM clearly shows the high-resolution AFM movies of retrograde actin flow on the plasma membrane, morphogenesis of filopodia, membrane ruffles, pit formation, and endocytosis in COS-7, HeLa cells and hippocampal neurons without any significant cellular damages for several tens of minutes. This is because the long AFM tip robustly steps over cells on the substrate without collisions between a cantilever and cells during HS-AFM scanning. Furthermore, the sharpness of the tip (~5 nm) provided lateral resolution of nanometers. This long-term stability of imaging with LT-HS-AFM permitted us to visualize morphological dynamics of cells in response to external stimuli such as drugs and ligands.

LT-HS-AFM also has limitations. Although LT-HS-AFM can image nanometer scale structural information, it does not provide the identity of the structure observed. Thus, the combination LT-HS-AFM with advanced optical microscopy could open the new window to cell biology. For example, by comparing the timing of endocytosis events in fluorescence image with LT-HS-AFM structural information, it is possible to identify the protein functions and cellular event. Further, combining LT-HS-AFM with fluorescence resonance energy transfer (FRET) imaging^39^ or optical nanoscopy techniques^40^,^41^ could add further information about molecular interactions and intracellular signal transduction.

In summary, we demonstrated the potential of LT-HS-AFM provides to directly visualize nanometer-scale morphological changes in live cells. In particular, the successful observations of nano-structural dynamics in live neurons will open the possibility of visualizing the morphology of plasticity of synapses at nanometer resolution in real time in the near future.

## Methods

### Fabrication of long AFM tips on original AFM tips of cantilevers

We used cantilevers measuring ~9 μm long, ~2 μm wide and ~0.13 μm thick with a spring constant of 100–200 pN/nm and ~0.8 μm tip at the end of the cantilever (BL-AC10DS, Olympus). To fabricate an additional tip on the original AFM tip by a phenol deposition technique^29^, we fixed the cantilever on a cylindrical chamber with small through-holes on its lid (~0.1 mm diameter) using both-side tapes (PELCO Tabs). In the holder ~10 mg of phenol crystal (Sigma) was included. Under a scanning electron microscope (Quanta 250, FEI), the focus of an electron beam was parked onto the cantilever tips at 30 kV, 5 mm working distance with 30 μm aperture size in a high-vacuum mode. A stylus-shaped deposition of amorphous carbon was produced at a growth rate of ~600 nm/min. The focused electron beam was irradiated for 1 min and repeated 5–7 times. The focus position was reset after each cycle to compensate for the mechanical drift of the instrument. After fabrication of EBD tips, cantilevers were further sharpened in the RF-plasma etcher chamber (PE-2000, South Bay Technology) filled with ~90% argon gas for ~10 s. Typical power of the RF plasma is about 10 W and the chamber pressure is ~50 mTorr. It reduced the apex radius of EBD tip to 5–8 nm. The AFM tip was used in 2–3 experiments until the tip accumulates contamination and the spatial resolution visibly degrades.

### Combined LT-HS-AFM and fluorescence microscopy setup

We built HS-AFM apparatus equipped with the wide-range HS-AFM scanner as reported^25^. We put the fabricated long-tip cantilever to HS-AFM for cellular imaging. The cantilever deflection was detected by detecting the position of a red laser beam (670 nm) reflected by the cantilever with a position-sensing quadrant photodiode^13^. The laser beam was focused onto a cantilever using a ×60 objective lens (CFI S Plan Fluor ELWD, Nikon). To combine the fluorescence microscopy, the excitation lamp (M470LM, Thorlab), dichroic filters (MDF-GFP, Thorlabs) and a CCD camera (DS-Fi2, Nikon) are installed under the HS-AFM set up (Fig. 1b). A cell transfected with mEGFP was located to the LT-HS-AFM scanning area (~40 × 40 μm^2^) using fluorescence imaging and subjected for nanometer imaging with LT-HS-AFM (Supplementary Fig. 1). HS-AFM images were acquired in tapping mode (100 nm free amplitude, ~800 kHz). LT-HS-AFM resolutions were set to 200 × 200 pixels^2^. The scan rate was set to 5 s per frame (40 lines/s) or 10 s per frame (20 lines/s). There was no obvious change in the spatial resolution during an experiment unless the tip was contaminated. The tip was brought to the sample at the speed of 1.5 μm/s using a motor (RK543AW, Orientalmotor) while the oscillation amplitude was monitored. When the oscillation amplitude became ~10% of the free amplitude, the motor was stopped and an imaging session was started^29^.

### Cell culture, transfection and LT-HS-AFM observation of COS-7 and HeLa cells

COS-7 cells (ATCC #: CRL-1651) and HeLa cells (ATCC #: CCL-2) were cultured in Dubelco's modified eagle medium (DMEM) (GIBCO) supplemented with 10% fetal bovine serum (FBS) (GIBCO) at 37°C in 5% CO_2_, and transfected with plasmids using Lipofectamine 2000 (Invitrogen) according to the manufacturer's instructions. Approximately 3–4 hours after transfection, cells were spread to a poly-L-ornithine (Sigma) coated AFM stage for at least 20 hours. LT-HS-AFM imaging were performed ~24 hours after transfection. The AFM stage is made of glass and shaped to be a hexagonal cylinder with 0.8 mm side length and 2.0 mm height. LT-HS-AFM imaging was performed in imaging solution containing 15 mM HEPES (pH 7.4), 142 mM NaCl, 5 mM KCl, 2 mM CaCl_2_, 2 mM MgCl_2_ and 25 mM glucose. The temperature of imaging solution was kept at 32–34°C by heating the LT-HS-AFM chamber. Imaging solution was perfused for every 10 min. In some experiments, cells were treated with 20 ng/mL cytochalasin D (Sigma), 20 μg/mL insulin (Sigma), 20 ng/mL epidermal growth factor (EGF) (Sigma) or 80 μg/mL dynasore (Sigma).

### Primary culture, transfection and LT-HS-AFM observation of hippocampal neurons

Dissociated hippocampal cultures were prepared from newborn Sprague Dawley rats at postnatal day 0–1, as described previously^38^. Briefly, the hippocampi were dissected in HBSS-HEPES (GIBCO), washed several times with HBSS-HEPES. Then, hippocampi were digested with papain (100 units, Worthington Biochemical) at 37°C for 15 minutes and gently pipetted in solution containing DNase I type IV (0.6 mg/mL, Sigma) until the tissue was mostly dissolved. Undissociated tissue was removed from the solution by passing the mixture through a cell strainer (40 μm, BD Biosciences). Dissociated neurons were collected in 50 mL conical tube and precipitated with centrifugation (1000 rpm for 5 min). The precipitate was resuspended in a culture medium consisting of Minimum Essential Medium (MEM) (GIBCO) and 10% FBS to various cell concentrations. The neurons were placed onto poly-L-lysine/laminin-coated AFM stage for 1 hour. The medium was replaced with a medium containing Neurobasal Medium (GIBCO), B-27 Supplements (GIBCO), and GlutaMAX (GIBCO) and cultured at 37°C in 5% CO_2_. Cultured hippocampal neurons were infected with lenti virus encoding synapsin promoted mEGFP at 2–3 days *in vitro*.

## Author Contributions

M.S. and R.Y. conceived and designed the experiments. M.S. and T.U. made the software for HS-AFM instrument. M.S. developed the LT-HS-AFM instrument. M.S. performed the all experiments. M.S. and R.Y. analyzed the data. T.A. and R.Y. supervised the experiments. M.S. and R.Y. wrote the paper. All authors discussed the results and commented on the manuscript.

## Supplementary Material

Supplementary Information Supplementary Information

Supplementary Information Long-tip high-speed AFM movie of the leading edge of a living COS-7 cell under a control condition (left) and after the application of cytochalasin D (20 ng/mL) (right).

Supplementary Information Long-tip high-speed AFM movie taken from the same area as in Supplementary Movie 1 after the washout of cytochalasin D.

Supplementary Information Long-tip high-speed AFM movie of the leading edge of a living COS-7 cell under a control condition (left) and after the addition of insulin (20 μg/mL) (right).

Supplementary Information Long-tip high-speed AFM movie of a living HeLa cell before (left) and after the application of EGF (20 ng/mL) (right).

Supplementary Information Long-tip high-speed AFM movie take from an area near the nucleus of a living COS-7 cell before (left) and the addition of dynasore (80 μg/mL) (right).

Supplementary Information Long-tip high-speed AFM movie of a living COS-7 cell after the washout of dynasore (same area as in Supplementary Movie 5).

Supplementary Information Long-tip high-speed AFM movie of a living COS-7 cell transfected with mEGFP-Rab5(Q79L).

Supplementary Information Long-tip high-speed AFM movie of a living cultured hippocampal neuron at 9 days in vitro (DIV).

Supplementary Information Long-tip high-speed AFM movie of a living cultured hippocampal neuron at 13 DIV.

Supplementary Information Long-tip high-speed AFM movie of a living cultured hippocampal neuron at 15 DIV.
