# Supplementary material for: Long-tip high-speed atomic force microscopy for nanometer-scale imaging in live cells
Source: Sci Rep. 2015 Mar 4;5:8724. doi: 10.1038/srep08724 (PMC4348644; doi:10.1038/srep08724)
Supplement: Supplementary Information [file srep08724-s1.pdf]

Supplementary Information

Long-tip high-speed atomic force microscopy for nanometer-scale imaging in live cells

Mikihiro Shibata[1, 2] *, Takayuki Uchihashi[3,4,5], Toshio Ando[3,4,5] & Ryohei Yasuda[1,2] *

[1]Max Planck Florida Institute for Neuroscience, Jupiter, FL 33458, USA.

[2]Department of Neurobiology, Duke University Medical School, Durham, NC 27710, USA.

[3]Department of Physics, Kanazawa University, Kanazawa 920-1192, Japan.

[4]Bio-AFM Frontier Research Center, Kanazawa University, Kanazawa 920-1192, Japan.

[5]CREST/JST, Tokyo 102-0075, Japan.

*Correspondence and requests for materials should be addressed to
M. S. (mikihiro.shibata@mpfi.org) or to R.Y. (ryohei.yasuda@mpfi.org)

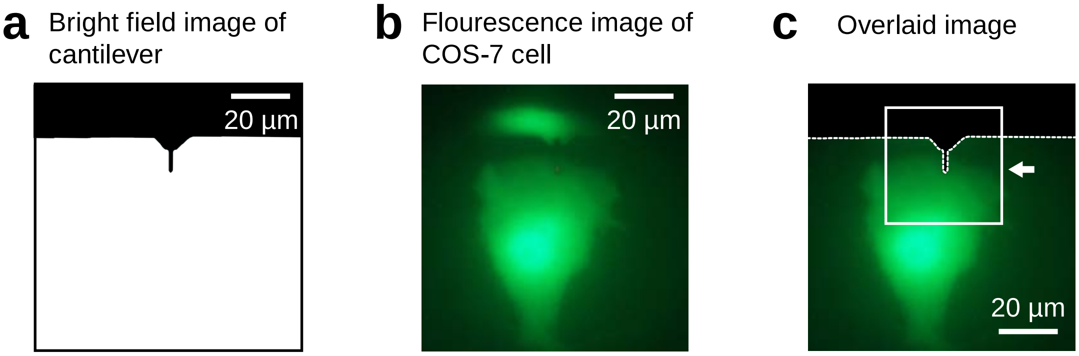

Supplementary Figure 1│ Combined LT-HS-AFM and fluorescence microscopy. (**a**) A bright field image of the cantilever. (**b**) A fluorescence image of a COS-7 cell transfected with mEGFP. The base of the cantilever causes a shadow on the fluorescence image. (**c**) An overlay image of the cantilever **a** and the fluorescence image **b**. The white dotted line displays the outline of the cantilever shadow. The white square with an arrow indicates the maximum LT-HS-AFM scanning area (~ 40 x 40 µm^2). The cell was manually placed in the LT-HS-AFM scanning area using micrometer heads, and then further refined the position with the piezo stage scanner.

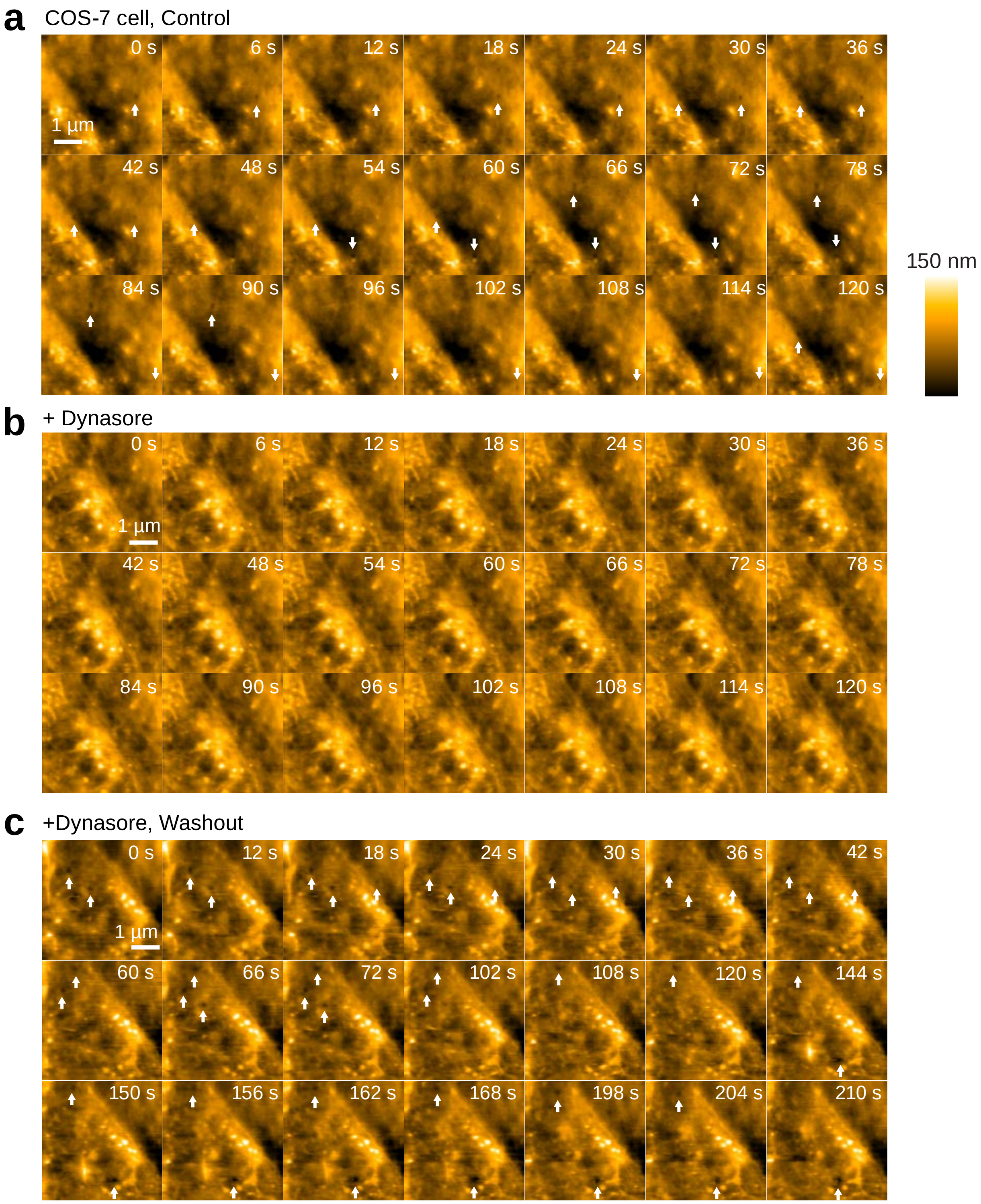

Supplementary Figure 2| LT-HS-AFM images of a living COS-7 cell in response to the application of dynasore. (**a**-**c**) A sequence LT-HS-AFM topographical images of the same cell as in **Fig. 3a** before **a**, the addition of dynasore (80 µg/mL) **b** and the washout **c**. White arrows indicate the pit formation. The corresponding movie (6 s per frame) is in **Supplementary Movies 5** and **6**.

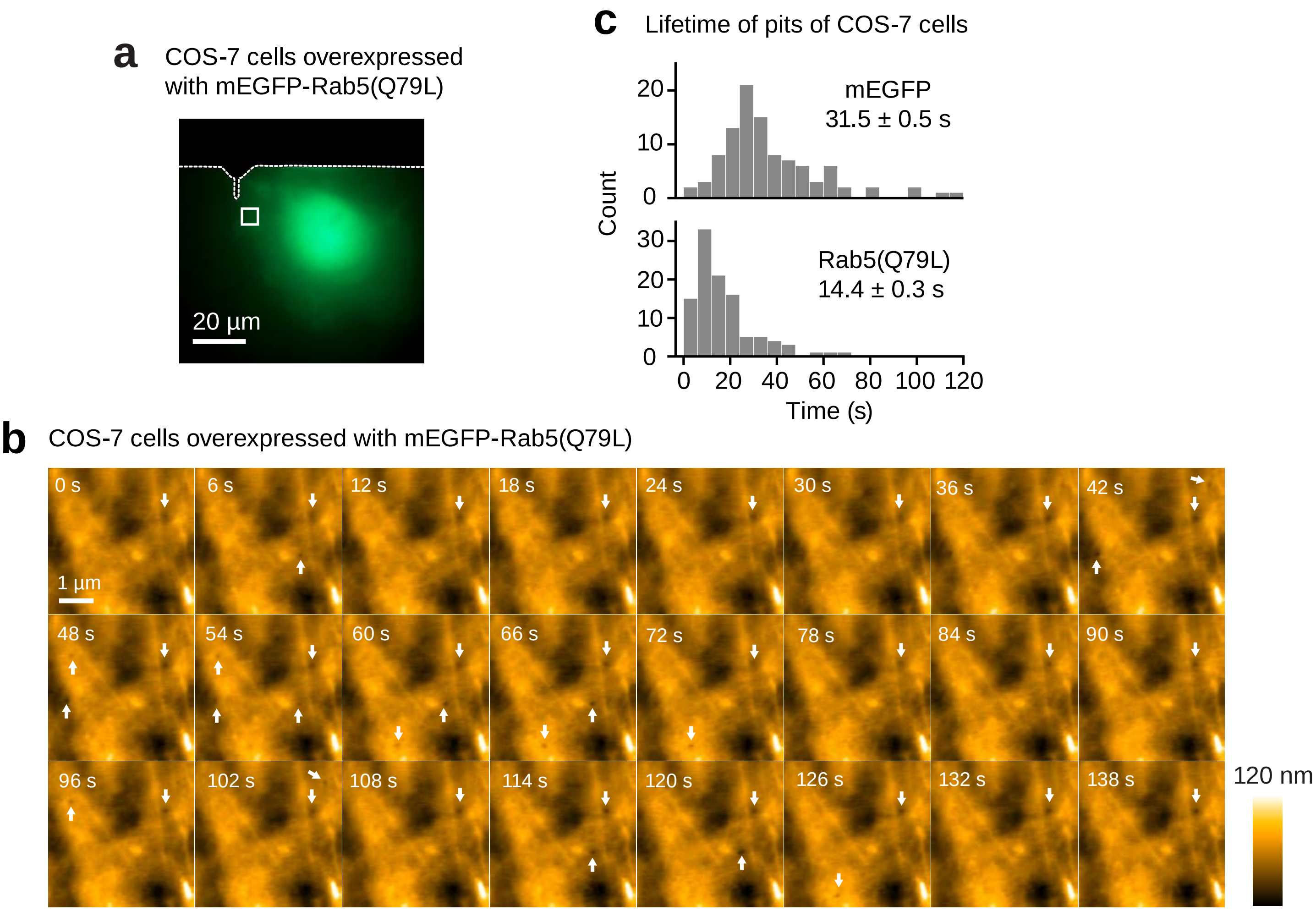

Supplementary Figure 3│ LT-HS-AFM images of a living COS-7 cell overexpressed with mEGFP-Rab5(Q79L). (**a**) Fluorescence image of a COS-7 cell transfected with constitutively active Rab5 [mEGFP-Rab5(Q79L)]. The white dotted line shows the shadow of the cantilever. The white square corresponds to the LT-HS-AFM imaging area. (**b**) A sequence of LT-HS-AFM topographical images of the same cell as in **a**. The white arrows indicate pit formations. The corresponding movie is in **Supplementary Movie 7**. (**c**) The histogram of the lifetime of pits for COS-7 cells transfected with mEGFP (upper) and mEGFP-Rab5(Q79L) (bottom). The number of total analyzed pits are 101 and 106 for COS-7 cells transfected with mEGFP and mEGFP-Rab5(Q79L), respectively (3 cells each).

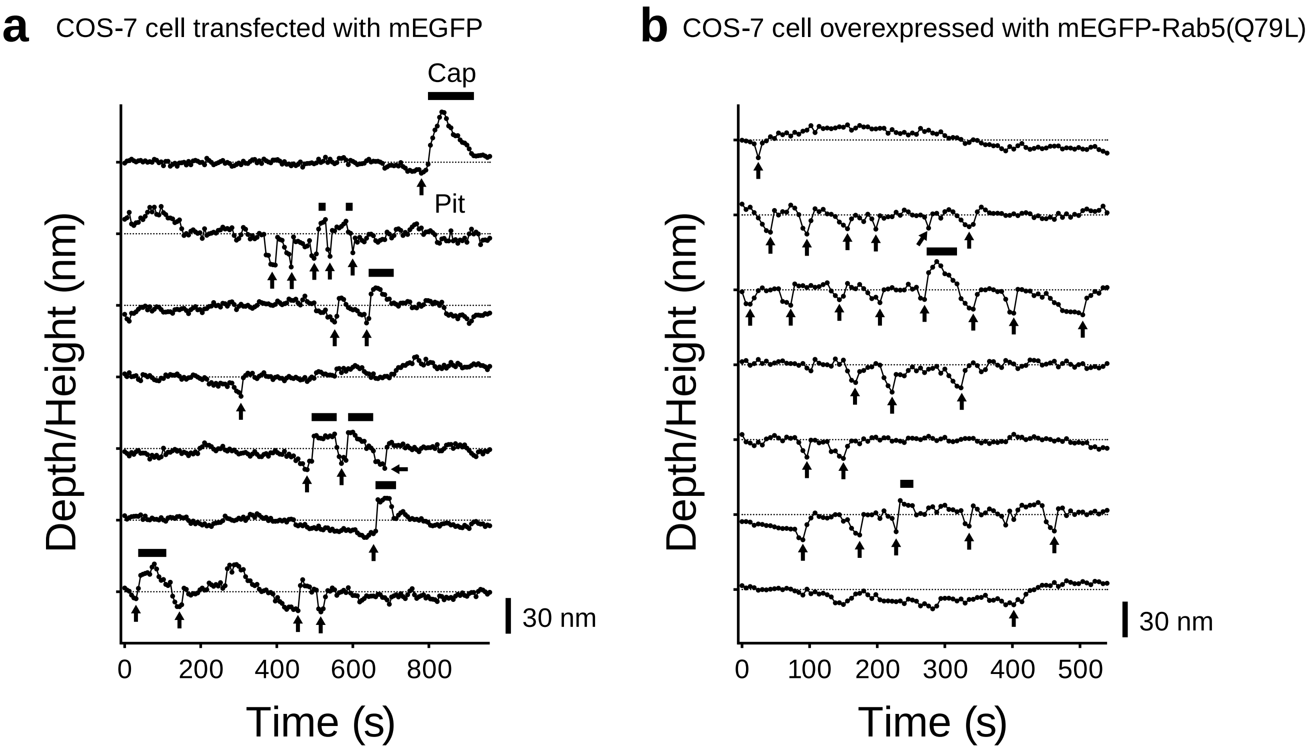

Supplementary Figure 4│Representative time courses of the depth of pits during the formation and the closure of pits in COS-7 cells transfected with mEGFP (a) and transfected with constitutively active Rab5 [mEGFP-Rab5(Q79L)] (b). The black bars indicate the formation of closure caps. Black arrows indicate formations of pits.

Supplementary Movie 1 l Long-tip high-speed AFM movie of the leading edge of a living COS-7 cell under a control condition (left) and after the application of cytochalasin D (20 ng/mL) (right). Under a control condition (left), formation and retraction of protrusions and movements of vesicles were observed. The membrane dynamics were inhibited by application of cytochalasin D (right). See **Supplementary Movie 2** for movies showing the same area after the washout of cytochalasin D. The scan range: 10 μm × 10 μm, the imaging rate: 10 second per frame, the total imaging time: ~15 min. This movie is played at ×160 speed.

Supplementary Movie 2 l Long-tip high-speed AFM movie taken from the same area as in **Supplementary Movie 1** after the washout of cytochalasin D. The membrane dynamics were recovered. The scan range: 10 μm × 10 μm, the imaging rate: 10 second per frame, the total imaging time: ~13 min. This movie is played at ×160 speed.

Supplementary Movie 3 l Long-tip high-speed AFM movie of the leading edge of a living COS-7 cell under a control condition (left) and after the addition of insulin (20 μg/mL) (right). The membrane ruffling was activated in response to insulin application. The scan range: 10 μm × 10 μm, the imaging rate: 10 second per frame, the total imaging time: ~12 min. This movie is played at ×160 speed.

Supplementary Movie 4 l Long-tip high-speed AFM movie of a living HeLa cell before (left) and after the application of EGF (20 ng/mL) (right). EGF accelerated the retrograde actin flow. The scan range: 10 μm × 10 μm, the imaging rate: 10 second per frame, the total imaging time: ~16 min. This movie is played at ×160 speed.

Supplementary Movie 5 l Long-tip high-speed AFM movie take from an area near the nucleus of a living COS-7 cell before (left) and the addition of dynasore (80 μg/mL) (right). Formations and closures of pits were indicated with white arrows. See **Supplementary Movie 6** for movies showing the same area after the washout. The scan range: 6 μm × 6 μm, the imaging rate: 6 second per frame, the total imaging time: ~17 min. This movie is played at ×60 speed.

Supplementary Movie 6 l Long-tip high-speed AFM movie of a living COS-7 cell after the washout of dynasore (same area as in **Supplementary Movie 5**). The movie displays the recovers of formations and closures of pits. The scan range: 6 μm × 6 μm, the imaging rate: 6 second per frame, the total imaging time: ~9 min. This movie is played at ×60 speed.

Supplementary Movie 7 l Long-tip high-speed AFM movie of a living COS-7 cell transfected with mEGFP-Rab5(Q79L). The movie displays repeated quick formations and closures of pits than a COS-7 cell transfected with mEGFP (**Supplementary Movie 5**). The scan range: 6 μm × 6 μm, the imaging rate: 6 second per frame, the total imaging time: ~9 min. This movie is played at ×60 speed.

Supplementary Movie 8 l Long-tip high-speed AFM movie of a living cultured hippocampal neuron at 9 days in vitro (DIV). The movie displays rapid filopodia extension and retraction. The scan range: 5 μm × 5 μm, the imaging rate: 5 second per frame, the total imaging time: ~24 min. This movie is played at ×100 speed.

Supplementary Movie 9 l Long-tip high-speed AFM movie of a living cultured hippocampal neuron at 13 DIV. The movie displays the sheet-like ruffling structure around the dendrite. The movie also displays the formation and closure of pit on the dendritic surface (white arrows). The scan range: 5 μm × 5 μm, the imaging rate: 5 second per frame, the total imaging time: ~8.5 min. This movie is played at ×100 speed.

Supplementary Movie 10 l Long-tip high-speed AFM movie of a living cultured hippocampal neuron at 15 DIV. The movie displays highly dynamic morphogenesis of the spine-like structure. The scan range: 5 μm × 5 μm, the imaging rate: 5 second per frame, the total imaging time: ~20 min. This movie is played at ×100 speed.
